# Implementation of a novel ultrasound training programme for midwives in Malawi: A mixed methods evaluation using the RE-AIM framework

**DOI:** 10.3389/frhs.2022.953677

**Published:** 2023-01-18

**Authors:** Alexandra C. Viner, Monica P. Malata, Medrina Mtende, Gladys Membe-Gadama, Martha Masamba, Enita Makwakwa, Catherine Bamuya, David Lissauer, Sarah J. Stock, Jane E. Norman, Rebecca M. Reynolds, Brian Magowan, Bridget Freyne, Luis Gadama, Sarah Cunningham-Burley, Linda Nyondo-Mipando, Effie Chipeta

**Affiliations:** ^1^MRC Centre for Reproductive Health, The University of Edinburgh, Edinburgh, United Kingdom; ^2^Centre for Reproductive Health, Kamuzu University of Health Sciences, Blantyre, Malawi; ^3^Department of Obstetrics and Gynaecology, Kamuzu University of Health Sciences, Blantyre, Malawi; ^4^Malawi Epidemiology and Intervention Research Unit, Lilongwe, Malawi; ^5^Faculty of Health and Life Sciences, Institute of Life Course and Medical Sciences, University of Liverpool, Liverpool, United Kingdom; ^6^Centre for Medical Informatics, Usher Institute, The University of Edinburgh, Edinburgh, United Kingdom; ^7^Faculty of Health Sciences, The University of Bristol, Bristol, United Kingdom; ^8^Centre for Cardiovascular Science, The University of Edinburgh, Edinburgh, United Kingdom; ^9^Borders General Hospital, NHS Borders, Melrose, United Kingdom; ^10^Faculty of Health and Life Sciences, Institute of Infection, Veterinary and Ecological Sciences, University of Liverpool, Liverpool, United Kingdom

**Keywords:** implementation, ultrasound, training, gestational age, Malawi, midwives

## Abstract

**Introduction:**

Despite recommendation that all women receive an ultrasound in pregnancy prior to 24 weeks', this remains unavailable to many women in low-income countries where trained practitioners are scarce. Although many programmes have demonstrated efficacy, few have achieved longterm sustainability, with a lack of information about how best to implement such programmes. This mixed-methods study aimed to evaluate the implementation of a novel education package to teach ultrasound-naive midwives in Malawi basic obstetric ultrasound, assessing its impact in the context of the Reach, Effectiveness, Adoption, Implementation and Maintenance (RE-AIM) framework.

**Methods:**

The study ran across six sites in Malawi between October 2020 and June 2021, encompassing three phases; pre-implementation, implementation and post-implementation. Twenty nine midwives underwent a bespoke education package with matched pre and post course surveys assessed their knowledge, attitudes and confidence and “hands on” assessments evaluating practical skills. Training evaluation forms and in-depth interviews explored their satisfaction with the package, with repeat assessment and remote image review evaluating maintenance of skills.

**Results:**

28/29 midwives completed the training, with significant increases in knowledge, confidence and practical skills. Adherence to the education package varied, however many changes to the proposed methodology were adaptive and appeared to facilitate the efficacy of the programme. Unfortunately, despite reporting approval regarding the training itself, satisfaction regarding supervision and follow up was mixed, reflecting the difficulties encountered with providing ongoing in-person and remote support.

**Conclusion:**

This programme was successful in improving trainees' knowledge, confidence and skill in performing basic obstetric ultrasound, largely on account of an adaptive approach to implementation. The maintenance of ongoing support was challenging, reflected by trainee dissatisfaction. By evaluating the success of this education package based on its implementation and not just its efficacy, we have generated new insights into the barriers to sustainable upscale, specifically those surrounding maintenance.

## Introduction

The World Health Organization (WHO) has regularly cited the need for improved estimates of gestational age, a fundamental component of obstetric and neonatal care, as a public health priority (1–3), not only to enhance clinical care, but to strengthen global reporting of pregnancy complications and to facilitate evaluation of context-specific interventions to improve outcomes. Although considered the most precise (4–7) way to date pregnancies, and recommended by the WHO (8), ultrasound remains unavailable to the majority of women living in low- and middle- income countries (LMIC), where gestational age is determined from either the last menstrual period (LMP) or abdominal palpation, both of which are substantially less accurate than ultrasound (9–11).

Accurate estimates of gestational age using ultrasound are required to facilitate the correct timing of antenatal care, the appropriate estimation of fetal growth and to permit the identification of pre-term birth, enabling the optimal timing of referral and intervention and ensuring this is delivered in the most suitable setting (6, 12, 13). Pre-term birth is the leading cause of mortality in children under 5, with < 50% of babies born under 32 weeks in low-income countries surviving, compared with nearly all babies born at that gestation in high-income settings (14). Without accurate estimates of gestational age, women risk missing out on essential interventions which may improve outcomes for their babies, for example magnesium sulfate or antenatal corticosteroids (15, 16), or indeed risk being exposed to unnecessary ones, a factor especially pertinent in low- and middle- income countries.

Scaled provision of ultrasound is challenging for a number of reasons, ranging from economical and geographical, to human factors and the ability of healthcare systems to accommodate these services (17–22). While the procurement and maintenance of ultrasound machines, obtaining an effective energy supply and ensuring security are all important (20, 23, 24), one of the most frequently cited barriers is the lack of trained practitioners (19, 20). Although many programmes have demonstrated efficacy in training healthcare workers to perform ultrasound examinations (25–32), few have become embedded within pre-existing systems and achieved longterm sustainability, perhaps in part due to a relative lack of information about how best to implement such programmes (33).

Guidelines from the Association of Obstetricians and Gynecologists of Malawi (AOGM) recommend ultrasound in a variety of different antenatal and intrapartum scenarios. However, despite no published data describing the coverage of antenatal ultrasound in Malawi, anecdotal evidence suggests this is only provided consistently at the central hospitals, reflecting a mis-match between the recommended standards of care and what can actually be provided. Again, the lack of trained practitioners remains a significant problem.

This mixed-methods study aimed to evaluate the implementation of a novel education package to teach ultrasound-naive midwives in Malawi basic obstetric ultrasound, assessing its impact in the context of the Reach, Effectiveness, Adoption, Implementation and Maintenance (RE-AIM) framework (34). RE-AIM was chosen specifically for a number of reasons: (i) For its focus on realist evaluations of what works, for whom, in what setting, (ii) its extensive prior use to evaluate the provision of skills training in both high- and low- income settings, and (iii) its familiarity to the study group.

## Methods

This study was undertaken as part of a “parent” study exploring what factors may influence the upscale of basic antenatal ultrasound in LMIC settings (PACTR202010788566263). It was carried out across 6 sites in Malawi, selected to encompass both urban and rural facilities. The pre-implementation phase began in October 2020 and lasted 14 weeks. Implementation took place for 4 weeks in January-February 2021 and the post-implementation phase extended for 18 weeks until the end of June 2021.

### Pre-implementation phase (14 weeks)

#### Development of education package

The education package was developed by a team of Obstetricians from Malawi and the UK and aimed to teach the midwives basic obstetric ultrasound, namely to identify the number of fetuses, confirm fetal viability and presentation and to determine gestational age by measurement of the fetal femur length. Specific consideration was given to implementation, for example how to facilitate ongoing supervision and mentorship and the programme also included a number of small group sessions encouraging the midwives to think about how they would incorporate scans into their routine practice. These activities were mapped to the specific aspects of the RE-AIM framework. The materials were piloted twice in early 2020 and these iterations were instrumental in shaping the final programme (35). In order to facilitate remote image review, we also developed a bespoke app to permit image transfer and messaging between the trainees and faculty.

#### Facility preparation and participant recruitment

The participating sites were all primary level facilities, serving populations of between 20,300 to 596,230. Three were in Blantyre, one in Lilongwe, and two in the northern district of Karonga. The number of staff providing antenatal care at these sites ranged from 6 to 23 and in order to preserve the continuity of subsequent scanning services while accounting for staff absence or relocation, we sought to train a minimum of four midwives per site. Midwives were invited to participate having been identified by their local District Nursing Officer (DNO) as a key provider of antenatal care to women at the participating facilities and engaged in service improvement.

Trainers were recruited based on pre-specified criteria. All must have received training in basic obstetric ultrasound, have at least 12 months experience of performing ultrasound independently and be confident to troubleshoot any problems the trainees may encounter. Having been provided with a comprehensive training manual, the faculty were orientated to the programme virtually as a result of the ongoing COVID-19 pandemic. This brief was delivered by the team responsible for the course development and included the content and delivery of the curriculum, how to facilitate the “hands on” sessions and how to assess the trainees according to standardized criteria.

Each site was provided with a new, locally sourced, DP-10 ultrasound machine (Mindray, Shenzhen, China) and consumables such as ultrasound gel and tissues. Training materials, including comprehensive printed handbooks for both trainees and trainers were also distributed to participating sites, along with copies of the context specific guideline.

To ensure adequate numbers of client volunteers were available to participate in the ‘hands on' sessions of the training programme, participating sites were encouraged to recruit women in advance and request they attend on specific days. Pregnant women were eligible to participate as client volunteers if they were over 18 years old, thought to be over 14 weeks gestation and able to provide informed written consent. All were provided with a small allowance to cover their travel expenses.

### Implementation phase (4 weeks)

#### Delivery of education package

**Figure 1:**
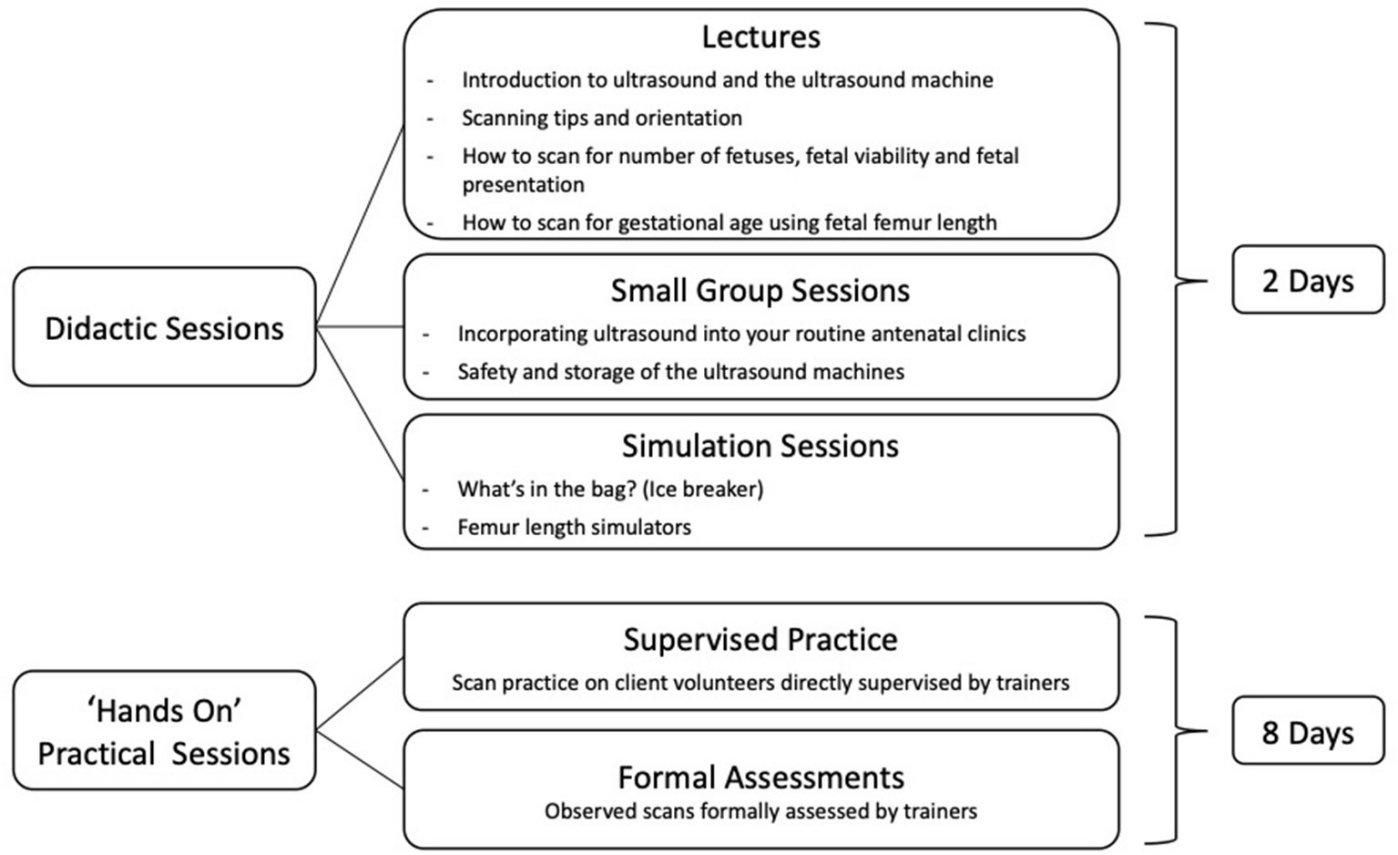
Overview of ultrasound training programme.

**Figure 2:**
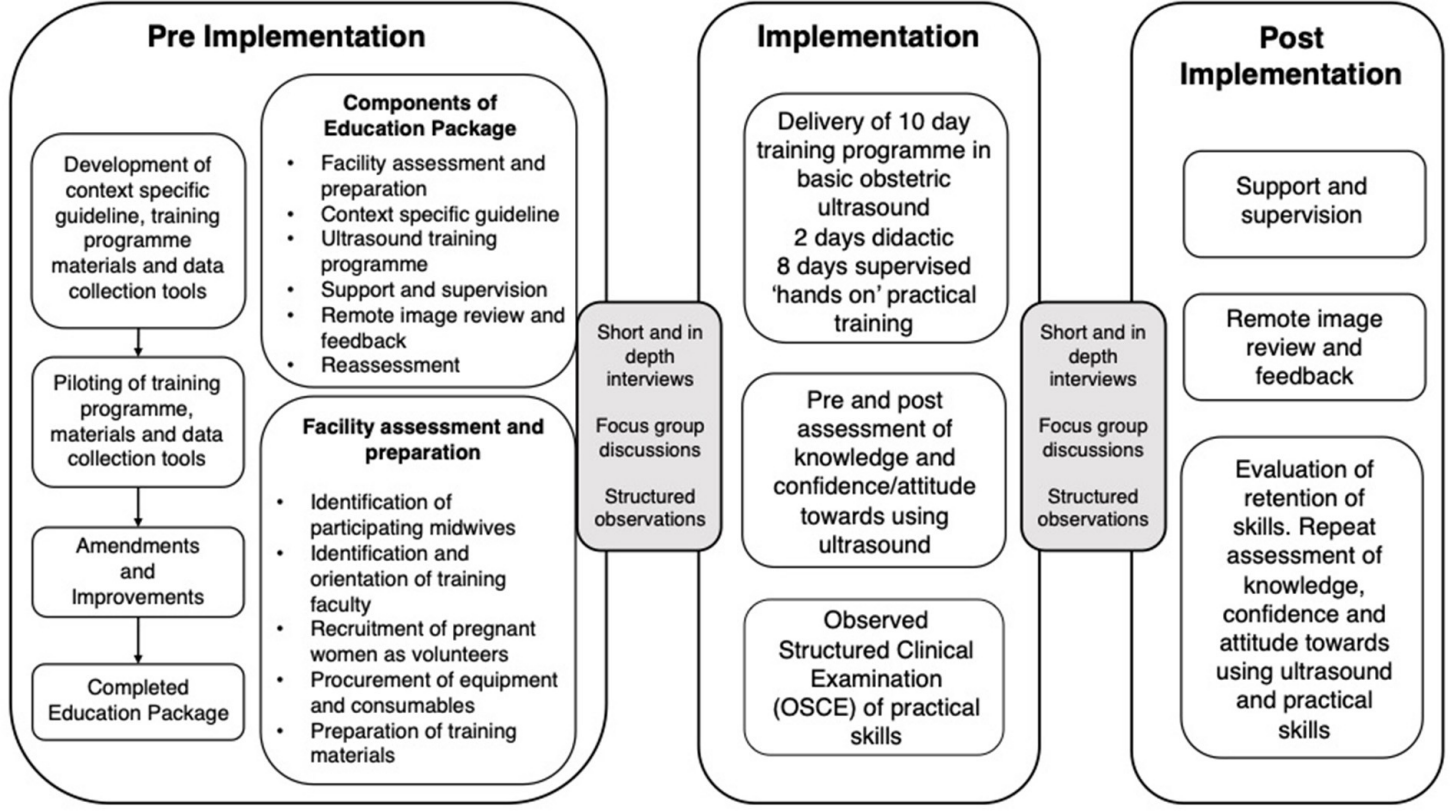
Overview of the implementation process.

The education package was delivered across the six sites in January-February 2021 with the ultrasound training programme consisting of 2 days didactic teaching, followed by 8 days of supervised “hands on” practice, as shown in Figure 1. The different components of the education package are detailed in Figure 2.

### Post implementation phase (18 weeks)

Remote supervision and image review was provided for 3 months following training. Trainees and faculty were requested to download our bespoke app to their personal devices and each was given an individual pin. Trainees were required to submit images which were assessed against pre-specified criteria by two independent reviewers experienced in obstetric ultrasound. Anonymised feedback was provided to all trainees every 1–2 weeks. Figure 2 provides an overview of the implementation process.

## Data collection and outcomes

As part of the training programme, each trainee undertook a 24-question multiple choice knowledge test to assess their theoretical understanding of ultrasound and completed a questionnaire using a 5 point Likert scale (1 = Strongly Disagree, 5 = Strongly Agree) to assess their attitude toward, and confidence using ultrasound. Both of these assessments were repeated immediately after completion of the training and again 3 months later. Trainees also undertook Observed Structured Clinical Examinations (OSCEs) to assess their practical skills and completed a post course evaluation form at the end of the programme. All data was collected on encrypted, password protected tablets and transferred to the secure study server daily. A proportion of purposively sampled midwives who had attended the training underwent an in-depth interview (IDI) at the end of the programme (*n* = 14, 48%) undergoing, with a smaller number (*n* = 6, 20%) of both trained and untrained midwives participating in focus group discussions (FGD). These took place both before and after the training and were significantly limited in number as a result of ongoing COVID-19 restrictions.

In-depth interviews and focus group discussions were undertaken by trained research assistants under the direct supervision of the authors MPM and CB. Sampling was purposive, targeting only the midwives who had undergone the ultrasound training and the interviews took place at the study sites until data was saturated. The board topic guide (Supplementary material) sought to explore the midwives response to training and the subsequent support they received, as well as their overall attitude toward using ultrasound. The objective of the focus group discussions was to better understand the impact of introducing the scan training on group dynamics within the individual sites.

Interviews and FGDs were conducted in either Chichewa or English, according to the participants' preference, and were recorded and transcribed verbatim. All transcripts were translated into English by trained transcribers, with verification undertaken by either MPM or CB. To ensure confidentiality, participants were dissuaded from using names and all recordings were stored using unique identifiers. Interviews were undertaken April-May 2021, approximately 10 weeks after the ultrasound training.

## Data analysis

Outcomes of this study were evaluated in the context of the RE-AIM framework, with Table 1 providing details of this process, along with outcome indicators and the relevant data sources.

**Table 1:** Implementation process outcomes and corresponding data sources.

**RE-AIM component**	**Indicators**	**Data sources**
Reach	Number of midwives participating and their Demographic/professional characteristics Number of clients recruited	Enrolment records
Effectiveness	Proportion of midwives certified as competent at the end of training Change in perception and confidence	Assessment scores Pre and post course questionnaires
Adoption	Motivation to attend training Acceptability of training	Post course evaluation form In-depth interviews
Implementation	Context and setting Education methodology Fidelity to education package and adaptions Cost	Facility assessments Education package Anecdotal reports of training In-depth interviews Administrative records
Maintenance	Strategy to ensure integration into pre-existing services and long-term provision of ultrasound Quality assurance/retention of skills	Protocol In-depth interviews Remote image review and repeat assessment scores

Quantitative data were analyzed using SPSS (IBM SPSS Statistics for Windows, Version 24.0. Armonk, NY: IBM Corp). Pre and post course questions evaluating similar concepts were allocated into 4 groups for analysis; perceived risk, beliefs regarding the role of ultrasound, confidence and attitudes toward using ultrasound, with reliability of the groupings tested using Cronbach's alpha. If scoring >0.8, good reliability, they were then analyzed as a group using a Wilcoxon signed rank test. If a group did not demonstrate adequate reliability, scoring < 0.8, the statements were analyzed as single items. Matched pre and post course knowledge tests were analyzed using paired *t*-tests. A *p*-value < 0.05 was considered statistically significant.

For the qualitative data, a thematic framework was generated based on the topic guides, with revisions undertaken as new themes emerged. Transcripts were iteratively coded using NVivo software (QSR International Pty Ltd), with codes grouped into categories and then themes. These were reviewed to identify patterns and make comparisons across the groups.

### Ethical considerations

As key stakeholders, the Ministry of Health in Malawi and the Directors of Health and Social Services (DHSS) of the participating sites were involved in the planning and implementation of this project. All participants (trainees and client volunteers) provided informed written consent, with participant information leaflets and consent forms available in both Chichewa and Tumbuka, as well as in English. This study was approved by the University of Edinburgh and the University of Malawi – College of Medicine Research and Ethics Committee (COMREC) P08/19/2768.

## Results

### Reach

Twenty nine midwives participated in the training, all of whom held either a Diploma or Degree in Nursing/Midwifery and none had any previous experience of using ultrasound. They had an average of 11 years of clinical experience, ranging from 1 to 30 years. All midwives who were invited to participate did so. All undertook practical assessments, with 22 completing matched pre and post course knowledge tests and 23 completing both pre and post course questionnaires. Post course evaluation data was available for 19 participants and 14 underwent an in-depth interview.

Training was provided by 15 trainers, all of whom held either a Medical Degree or a Diploma in Clinical Medicine or Radiology. Twelve had undergone formal training in ultrasound, with three receiving “on the job” training from experienced practitioners. They had an average of 6 years' experience in performing obstetric ultrasound, ranging from 1 to 16 years. Trainers were paid to contribute and all who were invited to participate did so.

Three hundred and ninety five pregnant women participated in the “hands on” element of the training, and of these 212 (54%) were unable to recall their LMP. Within the group who did know their LMP, a further 85 were assigned a new gestational age based on the measurements obtained by the trainer, suggesting that their LMP had been inaccurate. In total therefore, ultrasound improved the accuracy of pregnancy dating for 297 (75%) of the women who participated.

### Effectiveness

Twenty eight midwives achieved the criteria specified to “pass”; five consecutive scans where they achieved both a score of >65% on the OSCE and determined the gestational age to within +/- 7 days of the trainer, with one midwife unable to complete the programme due to illness. Knowledge improved after the training, (mean scores increased from 10.2 to 18) as did the midwives' confidence in performing ultrasound scans, as presented in Table 2. This quantitative increase in confidence was mirrored in the midwives' reflections on their progress.

“Though by and by, the more you practice the more you develop some experience and we tend to laugh ‘*ah ah the same patients we could spend so much time with but now we just go and follow the steps, nothing complicated*.' We have indeed gained some momentum.” FGD. Midwife from rural site K.

**Table 2:** Summary of quantitative results.

**Knowledge test (24 questions) *N* = 22 (Normal distribution)**	Mean scores (SD)		**Paired *t*-test**
	**Pre**	**Post**	***P* ≤ 0.0001 CI (6.51–9.21)**
	**10.2(3.3)**	**18(2.5)**	
Pre and post course questionnaire *N* = 23 (Non-normal distribution)– Beliefs around importance of ultrasound– Confidence performing ultrasound– Interest in performing ultrasound	Median scores		Wilcoxon signed rank test
	Pre	Post	
	5	5	*p* = 0.092
	2	5	*p* ≤ 0.0001
	5	5	*p* = 0.248
Practical assessments *N* = 405	Pass		Fail
	351		54

Although 13% (54/405) of the assessments were considered a fail, the incidence of this decreased as the training progressed. In eight cases failure was due to an inability to complete one of the five “critical tasks,” the most common problem being the inability to correctly determine presentation. In 41 cases it was due to insufficient accuracy in the determination of gestational age, and in five cases it was both.

### Adoption

#### Motivation to attend training

The most common reason the midwives chose to participate in the training, was that they believed skills in ultrasound would help them provide better care and also enhance their own skills. They further expressed their frustration at the limitations of trying to determine gestational age clinically.

“Most of the women do not know their last date of menstrual period so with the scanning introduced; in most cases we do find the EDD which most of the women did not know.” IDI with *Midwife from urban site Z*.

The post course questionnaire data (Table 2) supported an overall increase in the midwives' beliefs regarding the importance of ultrasound in antenatal care after training, although this result did not reach significance. The midwives were also motivated on a personal level, believing that gaining skills in ultrasound would be important for their professional development, although one expressed disappointment at not being paid extra to perform scans.

“This training is useful because it's like we are increasing our knowledge and our CV (Curriculum Vitae) is also updated.. this study is very beneficial in all levels, whether for the facility or individually.

” *IDI with midwife from rural site K*.“The investigators need to motivate healthcare staff by giving them something.. this is the only study that we are involved with without being paid anything.. healthcare staffs are not motivated to help.” *IDI with midwife from urban site Z*.

#### Acceptability of training

The midwives welcomed the concept of training in ultrasound, with 95% (*n* = 18/19) agreeing or strongly agreeing that the course was relevant to them. 100% (*n* = 19) reported that they had enjoyed the course.

“To us it is a welcomed idea… because now we have added another knowledge that we were not able to do… like a next step of caring for our patients.” *IDI with midwife from urban site Z*

Post course questionnaires revealed no difference in the midwives interest in performing scans (Table 2). A third of respondents to the post course evaluation (*n* = 6/19) requested the incorporation of additional components, for example liquor volume.

“The gap is there as we cannot scan pregnancy in the first trimester, of course I can just see a gestation sac but I cannot determine gestation age because we were given the limits… We should be taught all areas of scanning.” *IDI with midwife from urban site Z*.

Despite reporting satisfaction with the training course itself, attitudes toward the subsequent supervision was mixed, varying between sites. Some midwives viewed this favorably, whereas others felt the supervision had been lacking.

“Training was enough because we had supervisors who were helping us do the scanning properly and right now I would say we have support…” *IDI with midwife from urban site A*.

“We feel that the support being provided is very good from the people who taught us this intervention…” *IDI with midwife from urban site S*.

“…But we are somehow lacking supervision.” *IDI with midwife from rural site C*.

“Say maybe it is thirty percent. Cause I haven't been mentored but during the rotation for the other team, they were mentored once.” *IDI with Midwife from urban site Z*.

#### Usefulness of the training

Midwives all agreed that the provision of ultrasound scans was beneficial. They stated that they were able to provide better care by being able to accurately detect the EDD. Furthermore, they were also able to spot anomalies such fetal positioning, thus enabling them to respond in time.

“*What encourages women is that they want to know what is happening with their pregnancy. For example, sometimes the position of the child in the womb is abnormal, so it helps to identify that problem early and find ways to minimize complications during delivery.” IDI with midwife from urban site S*.

“*Scanning itself has improved quality of care. The clients, especially those that get scanned, are satisfied with our services because they feel that they have received the right care and this is encouraging to us. Again, since the introduction of scanning our job has been easier unlike in the past, with scanning we have a proper backing and when we are telling the woman her results we are so confident because it is backed up by visible evidence so as health workers scanning has really helped us in our job.” IDI with midwife from urban site A*.

### Implementation

#### Cost of implementation

The total cost of delivering this programme across six sites was £55,182. This included facility preparation, trainee and trainer allowances, client travel reimbursement, refreshments, all training materials and 6 new ultrasound machines.

#### Adaptations to implementation

As a result of COVID-19 restrictions, the theoretical component of the course was delivered virtually, with all trainees attending simultaneously. To enable trainers to rotate to the different sites, delivery of the “hands on” sessions were staggered. All trainees attended both the online theoretical and the practical “hands on” sessions, which, with the exception of the simulation sessions, were delivered according to the training manual. Importantly, a ratio of 1:4 trainers to trainees was maintained for all “hands on” sessions.

“From day one we were learning step by step until practicals. We were taught to follow an order of things.” *IDI with midwife from Urban Site A*.

Although all trainees completed practical assessments, the overall number of clients recruited per site varied greatly, affecting trainees' scanning exposure. The total number of scans performed by individual trainees ranged from 7 to 38, with an average of 18. Likewise, there were some issues with data collection, with not all trainees completing all forms. On one occasion this was due to an uploading error, however at other times it appears to have been oversight.

The greatest deviation from proposed methodology occurred in the provision of mentorship and follow up, which was purposed to occur as a hybrid of site visits and remote image review. Unfortunately, competing clinical duties, especially in the context of COVID-19, limited opportunities for the faculty to visit the sites and users with non-android devices were unable to download the bespoke app developed for remote review. Consequently, trainees were required to print a subset of their anonymised images, which were then collected in person with general and targeted feedback provided a week later.

#### Fidelity to training package

While the didactic and “hands on” elements of the training package were largely delivered as intended, the provision of supervision varied between the sites, with some deviating significantly from the intended processes.

### Maintenance

Aside from seeking refresher training and more robust mentorship, the midwives expressed strong sentiment that in order for ultrasound services to be sustained, efforts should be made to train more staff. Not only were they concerned about workload, but interruption to services when staff moved posts. They felt that the government should be involved in trying to facilitate this.

“For effective implementation of ultrasound scanning intervention, every health worker must be trained…” FGD with *midwives from rural site C*.

“It must involve every midwife because staff who are currently working at antenatal department won't be working there forever.” FGDs with *midwives from urban site A*.

“The main problem is workload because there are a lot of clients here but the nurses who provide scanning are few…” IDI with m*idwife from urban site A*.

“The NGO has specific time to support us so after those have left the government should take over so that the project is sustainable” IDI with *midwife from urban site S*.

They reported improved interpersonal relationships within the health facilities as a result of the introduction of ultrasound, feeling this to be a protective factor for longterm sustainability.

“The support being provided by our colleagues is good as well…” *IDI with midwife from urban site M*.

“When I fail on something I call a colleague for help.” IDI with *midwife from urban site Z*.

## Discussion

We have demonstrated that this bespoke education package was effective in improving the knowledge, confidence and practical skills of ultrasound-naive midwives in Malawi and that the recruited midwives were very motivated to participate in the training. Indeed, as 75% of the pregnant women who participated had either an unknown, or inaccurate gestational age, the potential benefit of initiatives such as this to enable midwives to improve pregnancy dating is clear. The midwives were largely satisfied with the programme, although there was some concern about the varied provision of mentorship and follow up. Implementation was challenging for a number of reasons, not least the COVID-19 pandemic, however many issues were overcome by the adoption of a flexible and pragmatic approach. A detailed description of the content and evaluation of training programme is provided elsewhere (35).

The “Reach” dimensions of the programme were largely satisfactory. Despite the midwives being a relatively senior cohort, this appeared to be a fair representation of the skill mix at the participating sites. While this may have influenced the time taken to develop practical skills, the fact this was variable within the group is very typical of training in ultrasound (36, 37). All midwives invited to participate did so, reflecting the interest in performing ultrasound scans which was also expressed during the qualitative interviews. Unfortunately, in order to preserve normal service provision at the health centers, not all midwives who worked there were able to attend. The diversity of the trainers, both in their background and experience, reflects the limited number of trained practitioners in LMICs who may available to facilitate such a programme (19, 20, 33), further demonstrated by the need for some to rotate around the sites to provide training. Although all trainers invited to participate did so, they were paid for their involvement which may have influenced their decision to contribute. Unfortunately, we do not have any information on the number of pregnant women who declined to participate and are therefore unable to comment on the proportion who were willing to undergo ultrasound, although this is being explored as part of the “parent” study.

In line with international recommendation, the midwives were assessed on their knowledge and understanding of obstetric ultrasound, as well as their practical skills (36). “Effectiveness” was demonstrated by improvements in the midwives' knowledge, confidence and practical skill in performing basic obstetric ultrasound, although satisfaction with the training was mixed. Differences in opinion were largely grouped by site, suggesting disparity in each site's “implementation” of the proposed training and follow up. Unfortunately, while being complicated by multiple connection issues, the virtual sessions also meant that the midwives missed out on the simulation sessions; a vital component of the training package which would have enabled them to familiarize themselves with both the machine and basic scanning techniques prior to scanning pregnant women (38–40). Some of the health centers suffered power outages during training, limiting practical experience, with others affected by local transport strikes, preventing the trainers from reaching the sites and therefore forcing sessions to be moved to different days. Despite recommending that pregnant women were approached in advance, recruitment of client volunteers to assist with the “hands on” sessions was very varied between sites, heavily impacting on trainees' individual exposure to practical experience.

The “Maintenance” dimension of this programme had mixed results. Plans for remote image review were complicated by users with non-android devices being unable to download the bespoke app and therefore being unable to submit or review images. Consequently, trainees were required to print a subset of their anonymised images, which were then collected in person, with feedback provided a few days later. Although sufficient, this caused delays in providing feedback and required the additional provision of consumables such as printer paper. Had our ethical approval permitted, an alternative option would have been to facilitate follow up via WhatsApp (41). Cheap and readily available, the use of WhatsApp in healthcare projects is well-established (42, 43) and as this is the current way in which midwives seek advice, it would be well aligned with the concept of embedding initiatives into pre-existing systems (44, 45).

Our qualitative findings regarding “adoption” are largely in keeping with the published literature regarding similar endeavors, with concerns about staffing, workload and the migratory workforce reflective of previous sentiments (19, 24, 46–48). Likewise, despite being motivated to perform ultrasound examinations, there is often resistance to being asked to do so without additional re-numeration (19, 20). Interestingly, this is the first study to suggest that the introduction of ultrasound may have a specific role in improving team dynamics.

Our study does have some limitations. Unfortunately, having to move some sessions to alternative days meant that post course tests/questionnaires were overlooked, limiting our matched pairs. Likewise, delayed timelines as a result of COVID-19, resulted in a lack of follow up data for all participants, therefore data pertaining to the retention of skills should be interpreted with caution. The strength of the qualitative data could have been improved by including formal structured observation of training sessions and in-depth interviews with the training faculty, however this was not possible within human resource constraints. Finally, the implementation of this education package was undertaken within the context of a research study and therefore it should be acknowledged that it will have benefitted from additional funds and support which may not have been available without substantial buy-in from the Malawian Government. That said, the progress of this programme has been endorsed by the Reproductive Health Directorate of the Ministry of Health of Malawi, the Society of Obstetricians and Gynecologists of Malawi and the Association of Malawian Midwives, with discussions ongoing about how to incorporate this into the nursing and midwifery curriculum in Malawi.

This package was strengthened by being delivered exclusively by local trainers, a contrast to many similar programmes and an important feature for sustainability. Not only were the local team better placed to understand the complexities of the setting, but also better equipped to troubleshoot any unexpected issues. A huge effort was made to adapt the training programme to try to mitigate the effects of COVID-19 and the scope for reactive and iterative changes within the protocol meant the approach to implementing the package was both pragmatic and representative of a “real world” scenario. By evaluating the implementation of this education package using the RE-AIM framework, we have been able to highlight the specific domains which require further input, in this case “maintenance.” In doing so we believe this will help focus efforts to optimize uptake and sustainability.

## Conclusion

By evaluating the success of this education package based on its implementation and not just its efficacy, we have generated new insights into the barriers to sustainable upscale, largely those of maintenance, which may provide important considerations for future scale up. While aspects of implementation were challenging, an adaptive approach meant that this education package was successful in improving the knowledge, confidence and practical skills of a representative cohort of ultrasound-naive midwives in Malawi. However, the subsequent maintenance of support and supervision was problematic, highlighting the need for more work to determine the optimum approach to facilitate this.

## Data availability statement

The raw data supporting the conclusions of this article will be made available by the authors, without undue reservation.

## Ethics statement

The studies involving human participants were reviewed and approved by University of Malawi College of Medicine Research and Ethics Committee P08/19/2768. The patients/participants provided their written informed consent to participate in this study.

## Diplomatic collaboration

Esmie Banda, James Boardman, Mia Crampin, Jean Desire Kabamba, Elizabeth Grant, Caroline Hollins Martin, Aisha Holloway, Khondwhani Kawaza, Corrine Love, Catherine Mkandawire, Patrica Munthali, Peter Mwaba, Shakira Namisengo, Everist Njelesani, Hilary Pinnock, Muriel Syacumpi, Frank Taulo.

## Author contributions

AV prepared the protocol for this study and with input and guidance from all authors. MPM, MMt, CB, SC-B, LN-M, and EC established the qualitative framework with AV, GM-G, MMa, BM, and LG developing the education package. GM-G, MMa, EM, and LG co-ordinated the delivery of the training in Malawi with MPM, MMt, CB, SC-B, LN-M, and EC overseeing the qualitative data collection and analysis. Quantitative data was analyzed by AV, who also prepared the first draft of the manuscript under the guidance of SS, JN, DL, BM, BF, LG, and EC. All authors provided critical insight for the manuscript.
