# Supplementary material for: Implementation of a novel ultrasound training programme for midwives in Malawi: A mixed methods evaluation using the RE-AIM framework
Source: Front Health Serv. 2023 Jan 18;2:953677. doi: 10.3389/frhs.2022.953677 (PMC10012721; doi:10.3389/frhs.2022.953677)
Supplement: Supplementary file 1 [file Data_Sheet_1.docx]

**TOPIC GUIDE FOR FOCUS GROUP WITH HEALTH CARE PROFESSIONALS**

**TIMEPOINT 2 – DURING INTERVENTION**

**LANGUAGE:** English

**STUDY: DIPLOMATIC (WP7)**

**AIM:** To scope health care professionals’ views of barriers and facilitators that are influencing implementation of ultrasound scanning into selected antenatal clinics in Malawi to accurately date gestational age of the infant and produce a more precise Estimated Date of Delivery (EDD).

**STAKEHOLDERS:** Doctors, Midwives, Sonographers

**MEMBER(S) OF INTERVIEW:**

|  | Name | Identification number | Role | Contact number |
| --- | --- | --- | --- | --- |
| 1 |  |  |  |  |
| 2 |  |  |  |  |
| 3 |  |  |  |  |
| 4 |  |  |  |  |
| 5 |  |  |  |  |
| 6 |  |  |  |  |
| 7 |  |  |  |  |
| 8 |  |  |  |  |

**DATE OF FOCUS GROUP/INTERVIEW:**

**TIME OF FOCUS GROUP/INTERVIEW:** Start_________ Finish_________ Length_________

**LOCATION OF FOCUS GROUP:**

**INFORMED CONSENT OBTAINED:** Yes / No

**NAME OF INTERVIEWER:**

**CONTACT NUMBER OF INTERVIEWER:**

**SEMISTRUCTURED INTERVIEW SCHEDULE (TOPIC GUIDE):**

**1. Introduction: overall experiences**

1.1 What are your overall views and experiences about how the introduction of ultrasound scanning is progressing?

1.2 Is everything proceeding as you expected?

**2. Barrier and enablers**

2.1 What do you think is working well/less well?

Probe: How do you think the scan is influencing the accuracy and value of EDD? How is this influencing management of care?

2.2 Are there any challenges with regards to infrastructure ? Do you have adequate space

2.3 Were there any challenges with training? Did you any experience because only a few midwives were trained? How has this affected your relationship with other midwives and service providers.

**3. From pregnant woman’s perspective**

3.1 What factors do you think support women to attend the antenatal clinic to have a scan?

3.2 What factors do you think are hindering women from attending the antenatal clinic to have a scan?

3.3 In your experience, how are women responding to the scan?

Probe any fears, positive responses e.g to receiving EDD, views of partners, families.

3.4 What reasons, if any, are women giving for refusing a scan?

**4. From health care professionals’ perspective**

4.1 What factors are supporting health care staff to implement ultrasound scanning?

Probe: what is helping them as individuals and what is helping their colleagues

4.2 What factors are hindering health care staff in implementing ultrasound scanning?

Probe: what is helping them as individuals and what is helping their colleagues

4.3 From your perspective, how are provider/client relationships being affected?

Probe: How do they feel talking with women about the scan and about expected date of delivery

**5. Health system**

5.1 What has been your experience of the education, training, and support being provided?

5.2 How is the introduction of ultrasound scanning affecting clinic logistics and flow?

5.3 Do you think there are any changes in how staff are working together?

**6. Recommendations**

6.1 From your point of view, what can maternity care staff do to encourage more women to attend the antenatal clinic to have an ultrasound scan?

6.3 Is there anything you would like to add to today’s discussion?

6.4 Do you have any further questions?

Our DIPLOMATIC project team would like to thank you for your valuable points of view. If you have any further questions or concerns please contact

___________________________________________________________________ at _________________________________________________________________
